# Clinical outcomes among HR+/HER2− metastatic breast cancer patients with multiple metastatic sites: a chart review study in the US

**DOI:** 10.1186/s40164-015-0023-0

**Published:** 2015-12-12

**Authors:** Jipan Xie, Yanni Hao, Nanxin Li, Peggy L. Lin, Erika Ohashi, Valerie Koo, Eric Q. Wu

**Affiliations:** Analysis Group, Inc., New York, NY USA; Novartis Pharmaceuticals Corporation, East Hanover, NJ USA; Analysis Group, Inc., Boston, MA USA

**Keywords:** Metastatic breast cancer, HR+/HER2−, Multiple metastases, Overall survival, Progression-free survival, Time on treatment

## Abstract

**Background:**

Hormone receptor-positive, human epidermal growth factor receptor-2-negative (HR+/HER2−) is the most common type of metastatic breast cancer (mBC). While mBC patients generally have poor prognosis with limited progression-free survival (PFS) and overall survival (OS), those with multiple metastatic sites may have even worse clinical outcomes due to multiple organ involvement. This study aimed to compare clinical outcomes including PFS, time on treatment (TOT), and OS between HR+/HER2− mBC patients with multiple metastases versus those with a single metastasis in a real-world clinical setting.

**Methods:**

This was a retrospective chart review study of postmenopausal HR+/HER2− mBC women who had failed a non-steroidal aromatase inhibitor in the adjuvant or metastatic setting and initiated a new treatment for mBC between 07/01/2012 and 04/15/2013. Patients were classified to one of two study groups (multiple metastases or single metastasis) based on the number of non-lymph-node metastases at the initiation of the new treatment. PFS, TOT and OS were compared between the two groups using Kaplan–Meier analyses and multivariable Cox proportional hazard models adjusting for patient disease and treatment characteristics. Separate Cox models were conducted including models with an interaction term between line of therapy and study group to assess the impact of multiple metastases on clinical outcomes across different lines of therapy.

**Results:**

A total of 699 patient charts were collected, including 291 patients with multiple metastases and 408 single metastasis patients. Worse performance status and a higher proportion of prior chemotherapy for mBC were observed among patients with multiple metastases. Overall, patients with multiple metastases had significantly shorter PFS [adjusted hazard ratio (HR) = 1.55, 95 % confidence interval (CI) 1.21–1.98], TOT (adjusted HR = 1.33, 95 % CI 1.05–1.67), and OS (adjusted HR = 1.77, 95 % CI 1.15–2.74) than single metastasis patients. Similar outcomes were observed in each line of therapy.

**Conclusions:**

Among HR+/HER2− mBC patients, patients with multiple metastases had significantly shorter PFS, TOT, and OS than single metastasis patients, highlighting the substantial clinical burden and unmet need for more efficacious treatments for the former group of patients.

## Background

Breast cancer (BC) is the leading cancer among women [1]. In the US, approximately 230,000 new BC cases were diagnosed in 2014 and 40,000 people died of BC in the same year [2]. The majority of patients are diagnosed at the early stage, for which treatment is generally effective, with a 5-year survival rate nearing 90 % [2]. However, BC also has a high recurrence rate, with up to 40 % of patients eventually progressing to metastatic BC (mBC) [3, 4]. In addition, around 5 % of BC cases are diagnosed at the metastatic stage [2]. Compared to early stage BC, mBC is associated with a much worse prognosis, with a 5-year survival rate of only 25 % [2].

MBC is categorized in several histological subtypes, based on the presence, absence, or overexpression of certain cell receptors [4, 5]. Hormone receptor-positive (HR+)/human epidermal growth factor receptor-2-negative (HER2−) mBC accounts for the majority of mBC cases [5]. Treatment guidelines for HR+/HER2− mBC recommend the use of three consecutive lines of endocrine therapy before the initiation of chemotherapy, or an earlier switch to chemotherapy in patients with visceral symptoms or rapidly progressing/life-threatening disease [6]. Recently, new targeted therapies are emerging and may revolutionize the treatment of mBC [7, 8]. For HR+/HER2− mBC in particular, everolimus in combination with exemestane had superior efficacy in extending progression-free survival (PFS) compared to exemestane alone in the BOLERO-2 trial [9]. Studies also suggest that everolimus is associated with longer PFS and time on treatment (TOT) in postmenopausal women with HR+/HER2− mBC [10, 11]. Palbociclib is another recently approved targeted therapy with demonstrated superior efficacy compared to endocrine therapy alone [12]. In addition, other targeted therapies with various mechanisms of action are under development, and some may become available in the near future.

With the rapid changes in the treatment landscape, the main question faced by physicians and payers is identifying the right patient population for these novel treatments with superior efficacies. Patients who have poor prognosis have a greater unmet need and may benefit more from these treatments. Several real-world studies have examined prognostic factors in mBC; among these, the number of metastases has been shown to be a significant predictor of overall survival (OS) [13–18]. However, these previous studies were all based on a single-center location and have generally focused only on OS [13–18]. While OS is an important outcome in mBC, other outcomes would also be of interest from a clinical perspective, such as PFS and TOT. In addition, a study covering different geographic regions in the US would be more informative and less subject to practices specific to individual centers.

To better understand clinical outcomes associated with multiple metastases, the current study was undertaken to compare PFS, TOT, and OS between HR+/HER2− mBC patients with multiple versus single metastases who were treated in community-based oncology practices in the US. The findings of this study could help us better assess the unmet need in HR+/HER2− mBC among patients with multiple metastases in the US. The study provided updated evidence on mBC populations with greater unmet needs that can inform decision-makers on potential targets for novel treatments in lieu of the rapidly changing landscape of mBC treatment.

## Results

### Baseline characteristics

A total of 188 physicians contributed 699 patient charts, including 408 patients with a single site and 291 patients with multiple sites of metastasis (Table 1). Physicians were mostly male (>70 %), practicing in a small/intermediate practice setting of 2–9 physicians (>70 %), and had more than 5 years of experience (>90 %). At index treatment initiation, the median patient age was 65 years for the multiple metastases group versus 64 years for the single metastasis group (Table 1). Both patient groups had similar median duration from initiation of the last adjuvant endocrine therapy to mBC diagnosis (19.2 vs. 18.9 months, respectively) (Table 1). The median follow-up duration from index treatment initiation was also similar between the two groups: 16.3 months among patients with multiple metastases and 16.7 months among patients with a single metastasis (p = 0.239) (Table 1).

**Table 1:** Patient baseline characteristics

Baseline characteristics^a^	Patients with multiple metastases	Patients with a single metastasis	P-value^†^
	N = 291	N = 408	
Age at BC diagnosis, median years (range)	61.0 (35.0, 83.0)	61.0 (33.0, 90.0)	0.785
Age at index treatment initiation, median years (range)	65.0 (38.0, 86.0)	64.0 (33.0, 91.0)	0.878
Menopausal status at the first BC diagnosis^b^, n (%)			
Postmenopausal	169 (58.1)	238 (58.3)	0.946
Race, n (%)			
White	155 (53.3)	254 (62.3)	0.017*
Non-white	136 (46.7)	154 (37.7)	
Insurance plan type at mBC diagnosis, n (%)			
Commercial/private insurance	151 (51.9)	217 (53.2)	0.460
Medicare only	115 (39.5)	166 (40.7)	
Others	25 (8.6)	25 (6.1)	
Type of index treatment, n (%)			
Endocrine therapy^c^	196 (67.4)	356 (87.3)	<0.001*
Chemotherapy^d^	95 (32.6)	52 (12.7)	
Line of index treatment, n (%)			
First line	81 (27.8)	206 (50.5)	<0.001*
Second line	97 (33.3)	97 (23.8)	
Third line and above	113 (38.8)	105 (25.7)	
mBC type, n (%)			
Recurrent patients with adjuvant endocrine therapy	184 (63.2)	290 (71.1)	0.033*
Recurrent patients without adjuvant endocrine therapy	39 (13.4)	54 (13.2)	
De novo	68 (23.4)	64 (15.7)	
Number of non-lymph-node metastatic sites at mBC diagnosis, n (%)			
1	58 (19.9)	228 (55.9)	<0.001*
2	75 (25.8)	132 (32.4)	
3	132 (45.4)	8 (2.0)	
4	20 (6.9)	2 (0.5)	
Sites of metastatic disease at mBC diagnosis, n (%)			
Bone	188 (64.6)	231 (56.6)	0.034*
Liver	110 (37.8)	53 (13.0)	<0.001*
Lung	151 (51.9)	90 (22.1)	<0.001*
Any visceral metastases^e^	228 (78.4)	143 (35.0)	<0.001*
Brain	3 (1.0)	2 (0.5)	0.654
Other	4 (1.4)	5 (1.2)	1.000
Number of non-lymph-node metastatic sites at index treatment initiation, n (%)			
1	0 (0.0)	374 (91.7)	<0.001*
2	208 (71.5)	0 (0.0)	
3	79 (27.1)	0 (0.0)	
4	4 (1.4)	0 (0.0)	
Sites of metastatic disease at index treatment initiation, n (%)			
Bone	224 (77.0)	231 (56.6)	<0.001*
Liver	177 (60.8)	47 (11.5)	<0.001*
Lung	215 (73.9)	87 (21.3)	<0.001*
Any visceral metastases^e^	288 (99.0)	139 (34.1)	<0.001*
Brain	10 (3.4)	2 (0.5)	0.005*
Other	6 (2.1)	2 (0.5)	0.073
Adjusted Charlson Comorbidity Index (CCI)^f^ at index treatment initiation, median (range)	0.0 (0.0, 8.0)	0.0 (0.0, 6.0)	0.002*
ECOG performance status at index treatment initiation, n (%)			
0	49 (16.8)	134 (32.8)	<0.001*
1	151 (51.9)	164 (40.2)	
2	45 (15.5)	24 (5.9)	
3	8 (2.7)	2 (0.5)	
Not recorded in medical record	38 (13.1)	84 (20.6)	
Use of chemotherapy for mBC before index treatment initiation, n (%)	66 (22.7)	42 (10.3)	<0.001*
Duration from initiation of last adjuvant endocrine therapy to mBC diagnosis, median months (range)	19.2 (0.0, 216.6)	18.9 (0.0, 130.8)	0.879
Duration from initiation of index treatment to available follow-up, median months (range)	16.3 (1.0, 25.2)	16.7 (1.0, 24.6)	0.239

*BC* breast cancer, *ECOG* Eastern Cooperative Oncology Group, *mBC* metastatic breast cancer

* p < 0.05

^†^ Statistical comparisons were conducted using Wilcoxon rank-sum tests for continuous variables and Chi square tests for categorical variables

^a^Disease characteristics described at new treatment initiation include age, treatment type, line of index treatment, number and sites of metastases, adjusted CCI, ECOG performance status, and prior chemotherapy for mBC. Menopausal status, race, insurance plan type, mBC type, number and sites of metastases, and time elapsed (months) from initiation of last adjuvant endocrine therapy to stage IV mBC diagnosis were assessed at mBC diagnosis. Duration from initiation of index treatment to available follow-up was assessed at the end of follow-up

^b^The exact variable for menopausal status was not collected in the study but instead imputed based on age (postmenopausal: age ≥60)

^c^Endocrine therapy includes endocrine monotherapy (anastrozole, exemestane, fluoxymesterone, fulvestrant, letrozole, megestrol acetate, tamoxifen), combination therapy with two endocrine agents (fulvestrant + anastrozole, fulvestrant + exemestane, fulvestrant + exemestane, fulvestrant + tamoxifen), and everolimus-based therapies (everolimus, everolimus + anastrozole, everolimus + exemestane, everolimus + fulvestrant, everolimus + letrozole)

^d^Chemotherapy includes chemotherapy monotherapy (capecitabine, docetaxel, gemcitabine, ixabepilone, paclitaxel, protein-bound paclitaxel, vinorelbine), combinational therapy of two chemotherapy agents (cyclophosphamide + docetaxel, cyclophosphamide + doxorubicin, doxorubicin + docetaxel, paclitaxel + gemcitabine), and combinational therapy of chemotherapy and endocrine therapy (anastrozole + paclitaxel, fulvestrant + capecitabine, fulvestrant + paclitaxel, letrozole + paclitaxel)

^e^Any visceral metastases refer to liver, lung, and other visceral metastases, including peritoneum, adrenal gland, and kidney

^f^The adjusted CCI calculates the comorbidity index excluding metastatic breast cancer (score of 6)

However, patients with multiple metastases had more aggressive mBC than patients with single metastasis. Specifically, at index treatment initiation, 77.0 % patients of the multiple metastases group had bone metastasis versus 56.6 % of the single metastasis group, liver metastasis was present in 60.8 versus 11.5 % of cases, lung metastasis was present in 73.9 versus 21.3 % of cases, and brain metastasis was present in 3.4 versus 0.5 % of cases, respectively (all p < 0.05) (Table 1). Multiple metastases patients also had a worse ECOG performance status than patients with single metastasis (ECOG ≥ 2 was recorded for 18.2 versus 6.4 % of patients, respectively, p < 0.001) (Table 1). Twice as many patients with multiple metastases had used prior chemotherapy for mBC than patients with a single metastasis (22.7 versus 10.3 %, p < 0.001) (Table 1).

### Progression-free survival (PFS)

**Fig. 1:**
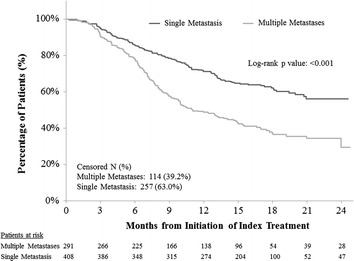
Comparison of progression-free survival between HR+/HER2− mBC patients with multiple metastases versus single metastasis

In the overall sample, patients with multiple metastases had significantly shorter PFS than single metastasis patients in both unadjusted (log-rank test p < 0.001, hazard ratio (HR) = 1.98, 95 % confidence interval (CI) 1.59–2.46, Fig. 1 and Table 2) and adjusted analyses (HR = 1.55, 95 % CI 1.21–1.98, Table 2). Results were consistent across different lines of therapy, though any differences between the two groups were not significant in the first-line setting (Table 3). In addition, second-line treatment (versus first-line), bone metastasis (versus no bone metastasis), and worse ECOG performance status were also associated with significantly shorter PFS (Table 2).

**Table 2:** Comparisons of progression-free survival, time on treatment, and overall survival, between patients with multiple metastases versus single metastasis

Parameters	Progression-free survival			Time on treatment			Overall survival		
	HR	(95 % CI)	P-value	HR	(95 % CI)	P-value	HR	(95 % CI)	P-value
Univariate analysis									
Multiple metastases (Ref: single metastasis)	1.98	(1.59,2.46)	<0.001*	1.78	(1.46, 2.18)	<0.001*	2.61	(1.78, 3.83)	<0.001*
Multivariable-adjusted analysis^a^									
Multiple metastases (Ref: single metastasis)	1.55	(1.21,1.98)	<0.001	1.33	(1.05, 1.67)	0.018*	1.77	(1.15, 2.74)	0.010*
Age at index therapy initiation	0.98	(0.97,1.00)	0.043*	0.97	(0.95, 0.99)	<0.001*	1.01	(0.98, 1.04)	0.646
Race (Ref: non-white)									
White	0.89	(0.71,1.12)	0.331	0.83	(0.67, 1.02)	0.074	0.88	(0.61, 1.29)	0.527
Insurance at mBC diagnosis (Ref: other or no insurance)									
Private	1.35	(0.85,2.14)	0.203	1.23	(0.81, 1.87)	0.326	1.12	(0.50, 2.50)	0.787
Medicare only	1.47	(0.89,2.42)	0.128	1.46	(0.93, 2.31)	0.103	1.27	(0.53, 3.04)	0.584
Type of index treatment (Ref: endocrine therapy^b^)									
Chemotherapy^c^	1.18	(0.91,1.55)	0.216	2.29	(1.80, 2.90)	<0.001*	1.84	(1.20, 2.81)	0.005*
Line of index treatment (Ref: first-line)									
Second-line	1.49	(1.06,2.09)	0.023*	1.75	(1.27, 2.41)	0.001*	1.26	(0.69, 2.29)	0.448
Third-line and above	1.34	(0.93,1.93)	0.114	1.18	(0.84, 1.65)	0.337	0.85	(0.44, 1.63)	0.618
mBC type (Ref: de novo)									
Recurrent with adjuvant endocrine therapy	1.27	(0.90,1.78)	0.175	1.53	(1.11, 2.12)	0.010*	1.40	(0.73, 2.64)	0.316
Recurrent without adjuvant endocrine therapy	0.40	(0.25,0.66)	<0.001*	0.45	(0.29, 0.71)	0.001*	1.06	(0.49, 2.31)	0.882
Adjusted CCI at index treatment initiation	1.08	(0.97,1.20)	0.155	1.09	(0.99, 1.21)	0.084	1.20	(1.02, 1.40)	0.024*
Bone metastasis at index therapy initiation									
(Ref: no bone metastasis)	1.56	(1.20,2.02)	0.001*	1.15	(0.91, 1.45)	0.233	0.85	(0.57, 1.27)	0.418
Performance status at index treatment initiation (Ref: ECOG 0)									
ECOG 1	1.44	(1.06,1.97)	0.021*	1.04	(0.79, 1.37)	0.785	1.76	(0.93, 3.32)	0.080
ECOG 2 and 3	2.60	(1.70,3.98)	<0.001*	1.87	(1.26, 2.78)	0.002*	5.04	(2.45, 10.40)	<0.001*
Unknown	1.77	(1.20,2.62)	0.004*	1.29	(0.90, 1.83)	0.166	3.15	(1.47, 6.72)	0.003*
Use of chemotherapy for mBC before index treatment initiation	0.82	(0.58,1.17)	0.283	0.89	(0.65, 1.23)	0.484	2.02	(1.15, 3.55)	0.014*
Duration from initiation of last adjuvant endocrine therapy to mBC diagnosis	1.00	(0.99,1.00)	0.374	1.00	(0.99, 1.00)	0.016*	1.00	(0.99, 1.01)	0.671

*BC* breast cancer, *CCI* Charlson Comorbidity Index, *CI* confidence interval, *ECOG* Eastern Cooperative Oncology Group, *HR* hazard ratio, *mBC* metastatic breast cancer, *Ref* reference

* P < 0.05; *Ref* reference group

^a^The model adjusted for age, race, insurance type at mBC diagnosis, treatment type, line of the index treatment, mBC type, adjusted CCI, bone metastasis at index therapy initiation, ECOG performance status, prior chemotherapy for mBC, and months from the initiation of the last adjuvant endocrine therapy to mBC diagnosis

^b^Endocrine therapy includes endocrine monotherapy, combination therapy with another endocrine agent, and everolimus-based therapies

^c^Chemotherapy includes chemotherapy monotherapy, combinational therapy of chemotherapy agents, and combinational therapy of chemotherapy and endocrine therapy

**Table 3:** Comparisons of progression-free survival, time on treatment, and overall survival between patients with multiple metastases versus single metastasis by line of therapy

Multiple metastases (Ref: single metastasis)	Progression-Free Survival			Time on Treatment			Overall Survival		
	HR	(95 % CI)	P-value	HR	(95 % CI)	P-value	HR	(95 % CI)	P-value
Univariate analysis									
First-line	1.87	(1.32, 2.65)	<0.001*	1.79	(1.30, 2.47)	<0.001*	3.32	(1.88, 5.85)	<0.001*
Second-line	2.01	(1.33, 3.03)	0.001*	1.83	(1.26, 2.65)	0.001*	3.29	(1.48, 7.33)	0.004*
Third-line and above	2.33	(1.54, 3.53)	<0.001*	2.04	(1.37, 3.02)	<0.001*	1.93	(0.94, 3.95)	0.074
Multivariate-adjusted analysis^a^									
First-line	1.43	(0.99, 2.08)	0.059	1.20	(0.85, 1.69)	0.313	1.98	(1.08, 3.64)	0.028*
Second-line	1.69	(1.10, 2.59)	0.017*	1.47	(0.99, 2.17)	0.055	2.41	(1.05, 5.52)	0.038*
Third-line and above	1.57	(1.00, 2.46)	0.049*	1.38	(0.90, 2.11)	0.143	1.09	(0.50, 2.36)	0.832

*CCI* Charlson Comorbidity Index, *CI* confidence interval, *ECOG* Eastern Cooperative Oncology Group; *HR* hazard ratio, *mBC* metastatic breast cancer, *Ref* reference

* P < 0.05

^a^The model adjusted for the following variables: age, race, insurance type at mBC diagnosis, treatment type, mBC type, adjusted CCI, bone metastasis at index therapy initiation, ECOG performance status, prior chemotherapy for mBC, and months from the initiation of the last adjuvant endocrine therapy to mBC diagnosis

### Time on treatment (TOT)

**Fig. 2:**
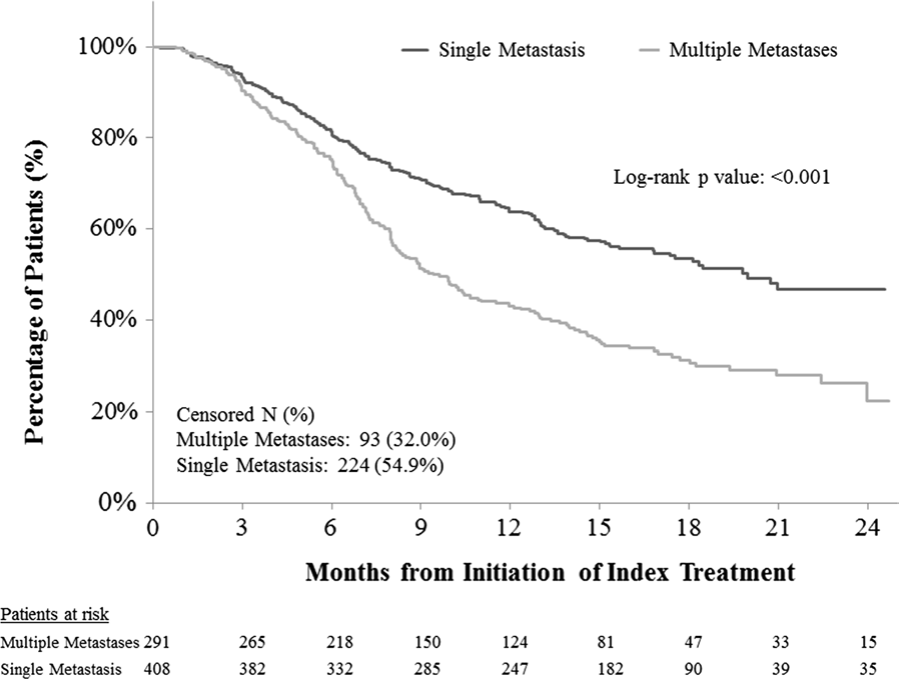
Comparison of time on treatment between HR+/HER2− mBC patients with multiple metastases versus single metastasis

Overall, patients with multiple metastases had significantly shorter TOT than single metastasis patients in both unadjusted (log-rank test p < 0.001, HR = 1.78, 95 % CI 1.46–2.18, Fig. 2 and Table 2) and adjusted analyses (HR = 1.33, 95 % CI 1.05–1.67, Table 2). Results across different lines resembled those in the overall sample, but were not significant (Table 3). Use of chemotherapy as index therapy, second-line treatment (versus first-line) and worse ECOG performance status were associated with significantly shorter TOT (Table 2).

### Overall survival (OS)

**Fig. 3:**
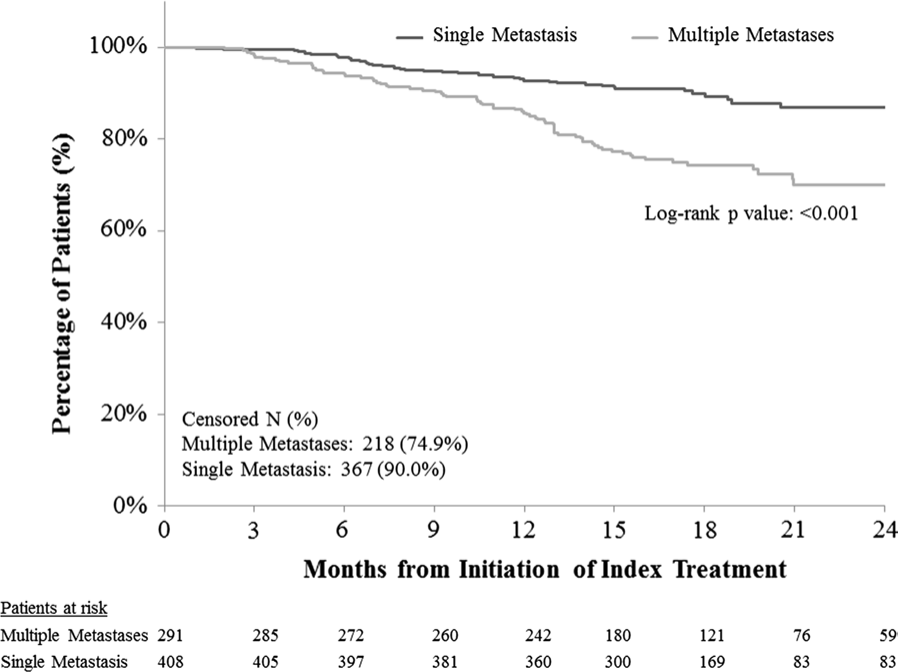
Comparison of overall survival between HR+/HER2− mBC patients with multiple metastases versus single metastasis

Overall, patients with multiple metastases had significantly shorter OS than single metastasis patients in both unadjusted (log-rank test p < 0.001, HR = 2.61, 95 % CI 1.78–3.83, Fig. 3 and Table 2) and adjusted analyses (HR = 1.77, 95 % CI 1.15–2.74, Table 2). Results across different lines also resembled those in the overall sample and were significant in the first- and second-line settings (Table 3). Use of chemotherapy as index therapy, higher adjusted Charlson comorbidity index (CCI), worse ECOG performance score, and prior chemotherapy for mBC were associated with significantly shorter OS (Table 2).

## Discussion

With advancements in the treatment of HR+/HER2- mBC, it is important to identify the patients who are in greater need of innovative therapies. Previous studies have identified multiple metastases as a risk factor for shorter OS. The current study added to the literature by using a national sample of mBC patients and comparing multiple clinical outcomes (PFS, TOT and OS) between patients with multiple metastases versus those with a single metastasis. The results demonstrate that patients with multiple metastases had significantly worse outcomes, as measured by PFS, TOT and OS, even after controlling for other factors, including bone metastasis. The findings are consistent across different lines of therapy. The current study confirms that the presence of multiple metastases is an independent prognostic factor of worse clinical outcomes among postmenopausal HR+/HER2- mBC patients. Furthermore, consistent with previous literature [14, 19–25], ECOG performance status was independently and significantly associated with all clinical outcomes examined in this study. Bone metastasis was significantly associated with PFS but not with other outcomes.

Due to multiple organ involvement, patients with multiple metastases are likely to be sicker and have more severe mBC than single metastasis patients [23, 26–28]. In the current study, patients with multiple metastases had a higher adjusted CCI score, worse ECOG performance status, and higher use of prior chemotherapy for mBC than single metastasis patients. The differences in clinical outcomes are quite striking between patients with multiple metastases and those with a single metastasis. The former group had a 55 % higher hazard of experiencing progression or death and a 77 % higher hazard of death, even after controlling for other potential prognostic factors of mBC (e.g., ECOG performance status). This impact was evident across different lines of therapy. These findings highlight the substantial burden and unmet need for more efficacious treatments in patients with multiple metastases.

In real-world practice, patients with multiple metastases are more likely to receive chemotherapy, often because their vital organs are affected and the patients are thus perceived to be in “visceral crisis” [29, 30]. However, chemotherapy is associated with serious side effects and can substantially impair patients’ quality of life [31, 32]. More importantly, chemotherapy has limited efficacy and effectiveness [11, 33]. These limitations call for more effective treatments to address the substantial unmet need of patients with multiple metastases.

Recently, a number of novel targeted therapies for the treatment of HR+/HER2− mBC have been approved or are in late-stage clinical development, including mammalian target of rapamycin (mTOR) inhibitors (e.g., everolimus), cyclin dependent kinase (CDK)-4/6 inhibitors (e.g., palbociclib), and phosphoinositide 3-kinase (PI3k) inhibitors (e.g., buparlisib). Large phase III randomized controlled trials (RCTs) have demonstrated the superior efficacy of these treatments compared to conventional endocrine monotherapies. For example, the median PFS associated with everolimus/exemestane combinational therapy was nearly three times longer than the PFS observed with exemestane monotherapy in the BOLERO-2 trial [9], and first-line palbociclib/letrozole combinational therapy doubled the PFS of patients receiving this treatment compared to patients treated with letrozole monotherapy in the PALOMA-1 trial [12]. In addition, everolimus-based therapy was also shown to be associated with significantly longer OS and PFS relative to endocrine therapy and chemotherapy [10, 11]. These innovative treatments may substantially improve outcomes among patients with multiple metastases. However, current literature suggests that individual treatment regimens are similar between first-line and subsequent lines of treatment [30, 33–36], and clinical guidelines have not recommended an optimal treatment sequence for the treatment of HR+/HER2− mBC [6, 37]. With a rapidly evolving treatment landscape, more studies are needed to assess the effects of newer treatments in this population and to shed light on the optimal sequencing of endocrine therapy, targeted therapy, and chemotherapy.

Adding to the previous literature, the current study could provide important evidence for decision-makers. From the physician perspective, identifying the patients who need more advanced treatment could facilitate a targeted approach to mBC management. For payers and health policy makers, understanding the burden of different subgroups of mBC could help in optimizing resource allocation for those in greater need of advanced treatment and could result in the improvement of the overall outcomes of mBC patients through a more cost-effective approach. Finally, the implications of more effective treatment go beyond extending PFS and OS. Such therapies may also improve the quality of life and productivity of patients. Therefore, the benefits of applying advanced treatments in this group with high unmet needs could be more substantial when viewed from the societal perspective.

This study has several limitations inherent to retrospective chart review studies. First, the results might be subject to selection bias if confounding factors were not identified or adjusted for in the analyses [38, 39]. In the current study, we adjusted for important baseline characteristics that were available in patient charts and which were thought to have the potential to affect clinical outcomes. Second, the frequency of follow-up could have differed between the two patient groups. Patients with multiple metastases had more severe mBC and may have been followed up more frequently than single metastasis patients. Thus, they may have been more likely to be identified as experiencing an event (e.g., progression) and this cohort may be biased against in the time-to-event analyses. Third, the current study did not collect any biopsy or histology data, which are important indicators to confirm mBC and inform treatment choice. Fourth, all patients were required to be postmenopausal at index treatment initiation based on eligibility criteria, but this study did not collect detailed menopausal status at BC diagnosis or mBC diagnosis. Despite these limitations, the current study provides important insights about real-world clinical outcomes for HR+/HER2− mBC patients with multiple metastases from a large nationwide sample, and could further help clinical and policy decision-making.

## Conclusion

Among HR+/HER2− mBC patients treated in community-based oncology practices in the US, those with multiple metastases had significantly shorter PFS, TOT, and OS than single metastasis patients, highlighting a substantial clinical burden and unmet need for more effective treatments for these high-risk patients.

## Methods

### Data source and patient selection

Community-based oncologists and hematologists who treated post-menopausal women with HR+/HER2− stage IV mBC (excluding patients with locoregional recurrences) were invited to participate in this chart review study from a nationwide online panel of over 9500 physicians practicing in the US. Participants were asked to randomly select up to ten eligible patients and enter relevant patient chart information into a secure electronic case report form (CRF). The CRF was developed by the study investigators and pilot tested with three physicians. No patient identification information was collected, and the study was approved by the New England Institutional Review Board.

Patients were eligible for this study if they had BC recurrence or progression on or after a non-steroidal aromatase inhibitor in the adjuvant or metastatic setting, and had initiated a new treatment for mBC, defined as the index treatment, between 07/01/2012 and 04/15/2013. Patients enrolled in any clinical trial or with a history of primary non-BC malignancy (with the exception of non-melanoma skin cancer and carcinoma in situ of the cervix uteri) during the 3 years prior to the first mBC diagnosis date were excluded. Patients were classified into two study groups based on the number of non-lymph-node metastatic sites at index treatment initiation: multiple metastases versus single metastases.

### Study outcome measures

PFS, TOT, and OS were assessed. PFS was defined as the time from index treatment initiation to disease progression or death, whichever occurred first. Progression was determined by the physician, based on radiographic evidence or tests, physical exams, symptoms, or other methods. TOT was defined as the time from index treatment initiation to discontinuation of the index treatment or death, whichever occurred first. OS was defined as the time from index treatment initiation to death from any cause. For all outcomes, patients who did not have an event at the end of the study were censored at the date of last follow-up.

### Statistical analysis

Patient baseline characteristics (i.e., before index treatment initiation) were compared between the two study groups using Wilcoxon rank-sum tests for continuous variables and Chi square tests for categorical variables. Patient characteristics included age, menopausal status (imputed based on age, postmenopausal: age ≥60), race, insurance type, type of index treatment (endocrine or chemotherapy), line of index treatment, mBC type, number of non-lymph-node metastases, sites of metastatic disease, adjusted CCI (excluding a score of six for metastatic cancer), ECOG performance status, prior chemotherapy for mBC, the time elapsed from the initiation of the last adjuvant endocrine therapy to mBC diagnosis, and duration from the initiation of the index treatment to end of follow-up.

PFS, TOT and OS were compared between patients with multiple and single metastases using Kaplan–Meier curves with log-rank tests and univariate Cox regression models. These outcomes were also compared between the two groups using multivariable Cox regression models adjusting for age at index treatment initiation, race, insurance type at mBC diagnosis, treatment type, line of the index treatment, mBC type, adjusted CCI, bone metastasis at index therapy initiation, ECOG performance status, prior chemotherapy for mBC, and time elapsed (months) from the initiation of the last adjuvant endocrine therapy to mBC diagnosis. In addition, separate Cox proportional hazard models were conducted, which included an interaction term between the line of therapy and study group in order to assess the impact of multiple metastases on clinical outcomes across different lines of therapy.

All analyses used a two-sided *p* value of 0.05 to determine statistical significance. Analyses were performed using SAS 9.3 (Cary, NC, USA).

## Abbreviations

BC: breast cancer
CCI: Charlson comorbidity index
CI: confidence interval
CDK: cyclin dependent kinase
ECOG: Eastern Cooperative Oncology Group
HR: hazard ratio
HR+: hormone receptor-positive
HER2-: human epidermal growth factor receptor-2-negative
mTOR: mammalian target of rapamycin
mBC: metastatic breast cancer
OS: overall survival
PI3k: phosphoinositide 3-kinase
PFS: progression-free survival
RCT: randomized controlled trials
TOT: time on treatment
